# Epidemiology of Neuroendocrine Neoplasms in the US

**DOI:** 10.1001/jamanetworkopen.2025.15798

**Published:** 2025-06-24

**Authors:** Arvind Dasari, Katrine Wallace, Daniel M. Halperin, Jessica Maxwell, Pamela Kunz, Simron Singh, Beth Chasen, James C. Yao

**Affiliations:** 1 Department of Gastrointestinal Medical Oncology, The University of Texas MD Anderson Cancer Center, Houston; 2 Department of Epidemiology and Biostatistics, University of Illinois, Chicago; 3 Department of Surgical Oncology, The University of Texas MD Anderson Cancer Center, Houston; 4 Department of Medicine, Section of Medical Oncology, Yale School of Medicine and Yale Cancer Center, New Haven, Connecticut; 5 University of Toronto, Toronto, Ontario, Canada; 6 Sunnybrook Odette Cancer Center, Sunnybrook Health Sciences Center, Toronto, Ontario, Canada; 7 Department of Nuclear Medicine, The University of Texas MD Anderson Cancer Center, Houston; 8 Winship Cancer Institute, Emory University, Atlanta, Georgia

## Abstract

**Importance:**

Neuroendocrine neoplasms (NENs) are increasing in incidence; prevalence and at the same time, practice patterns have also evolved, impacting classification and survival of these malignant neoplasms. However, updated epidemiological data are lacking.

**Objective:**

To define the epidemiological and survival trends of patients with NENs in the US.

**Design, Setting, and Participants:**

This cross-sectional study used data from the National Cancer Institute’s Surveillance, Epidemiology, and End Results (SEER) program on NEN cases from 1975 to 2021. Analysis of project data was conducted between August 2023 and August 2024.

**Main Outcomes and Measures:**

Annual age-adjusted incidence between 1975 and 2021, limited duration prevalence, and overall survival (OS) rates. Recent trends in survival were evaluated from 2000 to 2021 for the entire cohort as well as specific subgroups including distant stage gastrointestinal neuroendocrine tumors (NETs) and pancreatic NETs.

**Results:**

In this analysis of 145 447 NEN cases (mean [SD] age, 61.4 [14.7] years; 76 057 female [52.4%]), the age-adjusted incidence rate increased 5.2-fold from 1975 (1.64 per 100 000 persons; 95% CI, 1.43-1.87 per 100 000 persons) to 2021 (8.52 per 100 000 persons; 95% CI, 8.33-8.70 per 100 000 persons) except for a dip in 2020 likely related to the COVID pandemic. This increase occurred across all sites, stages, and grades but was most marked for localized stage neoplasms (13-fold; 1975: 0.40 per 100 000 persons [95% CI, 0.30-0.52]; 2021: 5.04 per 100 000 persons [95% CI, 4.78-5.30]), well-differentiated neoplasms (53-fold; 1975: 0.04 per 100 000 persons [95% CI, 0.02-0.09]; 2021: 2.30 per 100 000 persons [95% CI, 2.13-2.48]), and neoplasms with the appendix (12-fold; 1975: 0.11 per 100 000 persons [95% CI, 0.09-0.22]; 2021: 1.68 per 100 000 persons [95% CI, 1.39-1.78]) or rectum (12-fold; 1975: 0.11 per 100 000 persons [95% CI, 0.06-0.18]; 2021: 1.32 per 100 000 persons [95% CI, 1.19-1.46]) as primary sites. Since 2000 (SEER 17 registry), the sites with the highest incidence rates included lung (1.49 per 100 000 persons) and gastroenteropancreatic (GEP) NENs (6.1 per 100 000 persons); within GEP NENs, small bowel (1.4 per 100 000 persons) and pancreas (1.3 per 100 000 persons) were the most common sites. The estimated 20-year limited duration prevalence of NENs in the US on January 1, 2021, was 248 546 cases. OS for all NENs improved from the 2000-2006 period to the 2014-2021 period (hazard ratio [HR], 1.42; 95% CI, 1.38-1.45). In addition, other factors associated with survival included age, stage, grade, and primary site of origin. The median OS for all NENs was 11.8 years, and for distant-stage, well-differentiated neuroendocrine tumors it was 6.7 years with 10-year OS ranging from 17 410 patients (15.4%) with rectum as primary site to 17 505 patients (51.7%) with small bowel as primary site.

**Conclusions and Relevance:**

Incidence and prevalence of NENs continue to rise driven by increased diagnosis of early stage disease. Survival for NENs has also improved over time.

## Key Points

**Question:**

What are the epidemiologies of neuroendocrine neoplasms (NEN) in US?

**Findings:**

In this cross-sectional study evaluating 145 477 NEN cases in the US, age-adjusted incidence rates increased 5.2-fold between 1975 and 2021, with an annual percentage change of 3% between 2000 and 2020, and the 20-year limited duration prevalence projected in the US population on January 1, 2021, was 248 546. Survival for all NENs improved, including for patients with distant-stage gastrointestinal and pancreatic NENs.

**Meaning:**

These results suggest that NENs are increasing in incidence and prevalence; survival of patients with distant-stage disease has improved, likely reflecting improvements in therapies.

## Teaser

This cross-sectional study of neuroendocrine neoplasm cases in US adults examines epidemiological and survival trends.

## Introduction

Most neuroendocrine neoplasms (NENs) have indolent courses, and this coupled with their relative rarity mandate population-based registries such as the Surveillance, Epidemiology, and End Results (SEER) Program to delineate their epidemiology. The SEER program was initiated in 1973 and has since gathered data from population-based cancer registries.^1^ Since its inception (as SEER 8 registry), the SEER program has undergone several expansions, and the current (SEER 17) registry covers approximately 48% of the US population. Our last study on the epidemiology of NENs used data to 2014.^2^ Since then, several developments have occurred in the management of NENs, including approval of novel therapies such as peptide receptor radionuclide therapy, adoption of newer diagnostic modalities (DOTATATE-PET) and therapies (everolimus for extra pancreatic neuroendocrine tumors [NETs]), temozolomide-based therapies for pancreatic NETs, and telotristat treatment for carcinoid syndrome.^2,3,4,5,6,7,8^ The classification of gastroenteropancreatic (GEP) NENs has also been updated, while continuing to use indices of proliferation (Ki-67 and mitotic index) to categorize tumors into 3 grades, with grade 1 and grade 2 being well-differentiated NETs, grade 3 GEP NENs have been further subclassified morphologically into well-differentiated NETs (acknowledging their relatively indolent course similar to grade 1 and 2 NETs) and poorly differentiated neuroendocrine carcinomas (NECs). However, in contrast, lung NETs continue to be classified only into 2 categories—typical and atypical lung carcinoids—with the other more aggressive lung NECs being large or small cell lung carcinomas.^9^ Of note, since 2017, the SEER program has incorporated key NEN classification criteria including Ki-67 and mitotic index.^1^ In this study, we update the epidemiology of NENs using the latest SEER program data.

## Methods

### Data Sources and Clinical Characteristics

NEN cases diagnosed from 1975 to 2021 were obtained from the SEER incidence files (November 2023 release).^10^ Demographic information including race, ethnicity, and diagnosis information from the SEER database were complemented by population data from the US Census Bureau and mortality data from the US National Center for Health Statistics.^11,12^ Because the SEER stage criteria changed in 2004, we examined the years up to 2004 using the SEER historic stage variable and from 2005 to 2021 using the combined summary stage variable. Of note, a sensitivity analysis for the year that both variables were available (2004) generated near identical data.

Per MD Anderson institutional policy, no institutional review board approval was required; and no informed consent was needed because data were deidentified. Analysis of project data was conducted between August 2023 and August 2024. Results reporting adhered to Strengthening the Reporting of Observational Studies in Epidemiology (STROBE) reporting guideline.

### NEN Classification

Up to 2017, NEN cases were classified using the *International Classification of Diseases for Oncology, 3rd Edition*, as detailed and validated in prior publications.^2,13,14^ Briefly, we used the SEER histologic grade to classify NENs as grade 1 (G1), well differentiated; G2, moderately differentiated; G3, poorly differentiated; and G4, undifferentiated or anaplastic. G1 and G2 were combined to create a well differentiated (NET) category and G3 and G4 were combined into a poorly differentiated (NEC) category for analyses. Reporting of these data was discontinued in 2017.

After 2017, key criteria used for classification of NENs, ie, Ki-67 (measured by percentage) and mitotic count (per 10 high power fields [HPF]) were incorporated into the SEER registries. Per this classification, NENs grades were coded as G1 (mitotic count less than 2 and Ki-67 less than 3%); 2 (mitotic count 2 to 20 or Ki-67 3% to 20%) or 3 (mitotic count greater than 20 or Ki-67 higher than 20%). Separately, since 2017, the SEER registry also coded these neoplasms as well, moderately, or poorly differentiated, undifferentiated, or grade unknown. We used this information to provide descriptive data of NENs from 2018 to 2021. To avoid confusion and while awaiting further cross validation of these 2 different classification systems, analyses involving grade are limited to follow-up until 2017.

### Statistical Analyses

Incidence rates were calculated separately for the 3 registries: 1975-1991 (SEER 8), 1992-1999 (SEER 12), and 2000-2021 (SEER 17) databases. Incidence was calculated by stage at diagnosis, sex, and race. Prevalence was evaluated as 20-year limited duration rates. Using the SEER 8 registry, we also estimated the median overall survival (OS) by site, stage, and grade or differentiation with a maximum follow-up time of 30 years.

Three multivariable models were constructed using SEER 17 data: model 1 included the entire SEER 17 NEN cohort; models 2 and 3 were subsets that only included distant gastrointestinal and distant pancreatic NENs, respectively. We performed Cox proportional hazards multivariable analysis and hazard ratios (HRs) were calculated for overall mortality. The covariates known to influence NEN prognosis: grade or differentiation, race, age, stage, site, and time interval of diagnosis were included.

SEER*Stat software version 8.4.2 was used for incidence and limited-duration prevalence rates.^10^ The projected prevalence of NENs in the US population on January 1, 2021, matched by age, sex, and race, was calculated using ProjPrev version 1.0.6 (Data Modeling Branch, National Cancer Institute).^15^ Cox proportional hazards modeling was performed using SAS, v9.4 (SAS Institute Inc). Statistical significance was *P* < .05 in 2-sided tests.

## Results

The data had 145 447 NEN cases (mean [SD] age, 61.4 [14.7] years)—5514, 9155, and 130 778 in SEER 8, 12, and 17 registries, respectively. Of these, 76 057 (52.4%) were women (eTable 1 in Supplement 1).

### Annual Incidence

**Figure 1.**
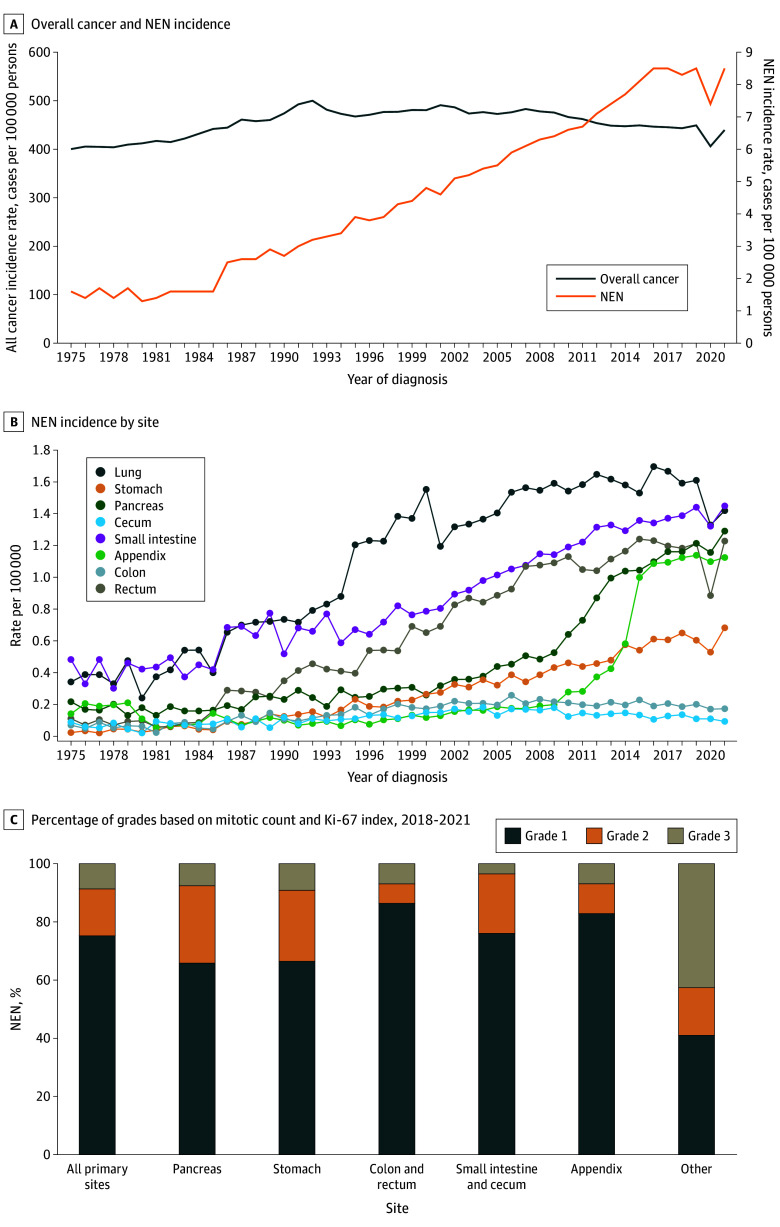
Incidence Trends From 1975 to 2021

Annual age-adjusted incidences of NENs and all malignant neoplasms were compared. The annual age-adjusted incidence (per 100 000 persons) of NENs was 1.64 (95% CI, 1.43-1.87) in 1975 and steadily increased to a peak of 8.52 (95% CI, 8.33-8.70) by 2021 except for a slight dip to 7.41 (95% CI, 7.37-7.72) in 2020, likely due to the COVID-19 pandemic (rates normalized in 2021 to prepandemic level) (Figure 1A). The annual percentage change for age-adjusted incidence from 2000 to 2021 in the SEER 17 registry was 2.9% (95% CI, 2.5%-3.4%; *P* < .001).

The mean (SD) age at diagnosis was lowest for NETs with primary sites in the appendix (47.1 [19.1] years) or rectal (55.8 [11.6] years) primary sites and localized disease stage (58.5 [14.9] years). Age-specific incidence rates from 1975 to 2021 were calculated for 3 age groups: under 50 years, 50 to 64 years, and 65 years and older. The most dramatic increases in incidence were noted in patients aged 50 to 64 years and 65 years or older with 5-fold increases to 17.61 and 28.44 per 100 000 persons, respectively. (Table 1; eTable 1 in Supplement 1).

**Table 1.** Incidence and Distribution of NETs by Age, Sex, and Race in the SEER 17 Registry

2000-2021	Age, mean (SD), y	Sex, rate per 100 000			Race, rate per 100 000				Sex, %		Race, %				
		All cases	Male	Female	White	Black	Asian	American Indian	Male	Female	White	Black	Asian	American Indian	Unknown
All cases	61.4 (14.7)	6.9	7	6.9	6.8	9.3	4.4	3.7	47.4	52.6	77.8	13.5	6.4	0.6	1.8
Disease stage															
Localized	58.5 (14.9)	2.9	2.7	3.1	2.7	4.2	2.3	1.7	31.2	35.6	31.8	37.3	42.6	38.1	42
Regional	62.8 (13.9)	1.1	1.1	1.1	1.2	1.3	0.5	0.5	13.5	12.7	14	11.2	9.1	9.3	3
Distant	64.8 (12.9)	1.3	1.5	1.2	1.4	1.6	0.7	0.7	18.1	15.2	17.6	14.2	13.0	12.9	1.5
Unstaged	61.2 (15.1)	0.8	0.9	0.8	0.8	1.3	0.5	0.5	37.3	36.6	36.6	37.3	35.3	39.7	53.5
Primary tumor site															
Lung	64.7 (13.5)	0.6	0.4	0.7	1.6	1.3	0.6	0.7	22.4	30.1	29.9	16.1	14.8	17.2	5
Cecum	63.2 (13.2)	0.1	0.1	0.1	0.1	0.2	0.1	0.1	2.5	2.6	2.7	2.6	0.9	2.4	0.4
Appendix	47.1 (19.1)	0.5	0.5	0.6	0.6	0.4	0.2	0.3	7.3	8.8	9	4.4	4.8	8.8	7.4
Stomach	62.5 (13.6)	0.5	0.4	0.5	0.5	0.6	0.3	0.4	6.5	9	7.9	7.2	7	11.8	10.4
Rectum	55.8 (11.6)	1.0	1.1	1.0	0.7	2.2	1.5	0.7	19.7	17.4	13.3	30.6	42.9	29.5	56.1
Small intestine	64.0 (13.0)	1.2	1.4	1.1	1.2	2.1	0.4	0.5	22.7	18.3	20.9	24.3	9.4	14.6	10.9
Colon	62.4 (13.7)	0.2	0.2	0.2	0.2	0.3	0.1	0.1	4.1	3.2	3.4	4.6	3.7	4.4	5.8
Pancreas	60.9 (13.9)	0.8	0.9	0.7	0.8	0.9	0.7	0.4	15	10.7	13.1	10.2	16.6	11.4	3.9

There were increases in the incidence of NENs from 1975 to 2021 across nearly all sites, stages, and grades. The largest increases in incidence over time were 12-fold in the appendix (1975: 0.11 per 100 000 persons [95% CI, 0.09-0.22]; 2021: 1.68 per 100 000 persons [95% CI, 1.39-1.78]) and the rectum (12-fold; 1975: 0.11 per 100 000 persons [95% CI, 0.06-0.18]; 2021: 1.32 per 100 000 persons [95% CI, 1.19-1.46]); since 2000 (SEER 17, 2000-2021), the largest increases were in the pancreas (4.3-fold) and appendix (12-fold) (Figure 1B). In SEER 17, the highest incidences were 1.40 per 100 000 persons in the lung, 4.27 per 100 000 persons in gastroenteropancreatic sites (1.41 per 100 000 persons in the small intestine, 1.31 per 100 000 persons in the pancreas, 1.22 per 100 000 persons in the rectum, and 1.20 per 100 000 persons in the appendix).

Among stage groups, incidence increased the most in localized NETs (14-fold) and among grade groups, incidence increased significantly most in well-differentiated NETs, from 0.1 per 100 000 persons in 1975 to 4.3 per 100 000 persons in 2017 (*P* < .001) (eFigure in Supplement 1). Of 44 733 NENs with a known grade between 2000 and 2017, 36 618 were well differentiated (81.9%; G1, 28 785 cases [1.6 per 100 000 persons]; G2, 7833 cases [0.4 per 100 000 persons]) and 8115 were poorly differentiated (18.1%; 0.5 cases per 100 000 persons). We also evaluated 19 529 NENs diagnosed between 2018 and 2021 who received a grade based on mitotic count and Ki-67 index score, of which 14 996 were G1 (76.8%), 3205 were G2 (16.4%), and 1328 were G3 (6.8%). Most common GEP sites for grade 3 NENs included colon and rectum (29%), pancreas (23%) and appendix (23%). Separately, histological differentiation was known only in 3456 NEN cases of which 3078 (89.1%) were well-differentiated, 163 (4.7%) were moderately differentiated, and 215 (6.2%) were poorly or undifferentiated. However, since the provided data do not allow further subclassification of G3 NENs into well- and poorly differentiated NENs, this analysis was not conducted (Figure 1C). Of 114 576 NENs with a known stage, 61 354 were localized (2.1 cases per 100 000 persons), 23 849 were regional (0.9 cases per 100 000 persons), and 29 373 were distant (1.2 cases per 100 000 persons) at the time of diagnosis.

Distant stage and primary site of small intestine and pancreas were more common in men while lung primary was more common in women. NET incidence rate was highest with Black patients (9.3 per 100 000 persons) and lowest in American Indian and Alaska Native patients. Lung was the most common primary site in White and American Indian and Alaska Native patients while rectum was the most common primary site in Black and Asian American or Pacific Islander patients (Table 1).

### Prevalence

**Figure 2.**
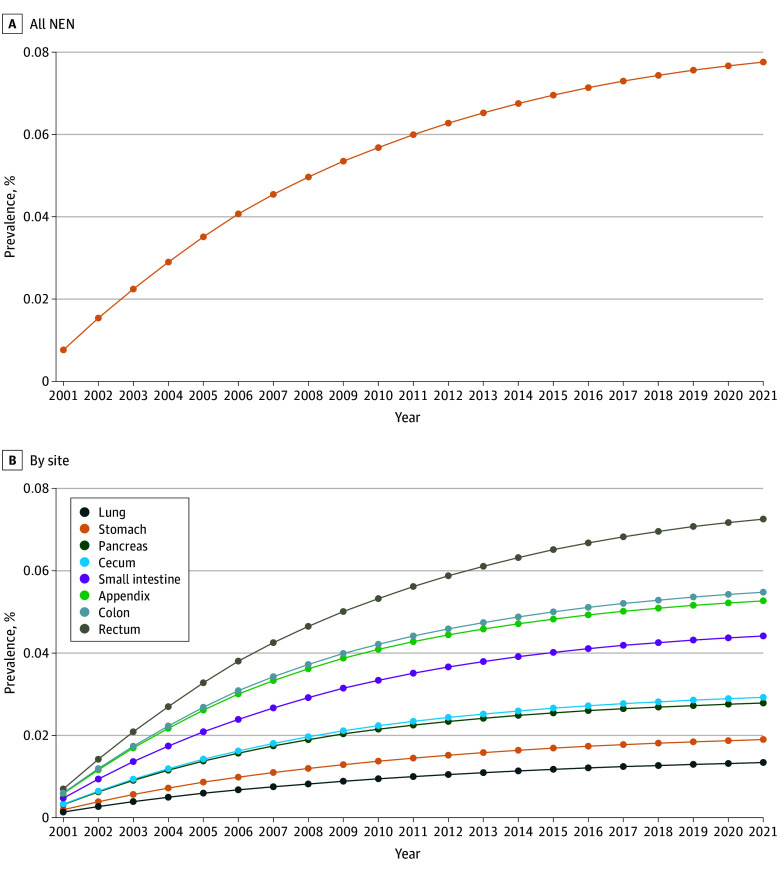
Limited Duration Prevalence of Neuroendocrine Neoplasms (NEN)

The 20-year limited-duration prevalence increased substantially, from 0.01% in 2000 (0.008%; 95% CI, 0.007%-0.008%) to 0.08% in 2021 (0.078%; 95% CI, 0.0003%-0.077%) (*P* < .001). The 20-year limited duration prevalence was highest in the rectum, followed by the small intestine and lung (Figure 2). The 20-year limited duration projected prevalence of NENs in the US population on January 1, 2021, was 248 546 persons matched by age, sex, and race (eTable 2 in Supplement 1).

### Survival

The median OS for all NENs was 11.8 years. As to be expected, localized NENs had better median OS (more than 30 years [to provide more user-friendly results for physicians, we divided months of survival by 12 and reported median years of survival]) compared with regional NENs (13.3 years) or distant stage NENs (1.2 years). Of those with known grades, well differentiated NETs had dramatically better median OS (16.8 years) compared with poorly differentiated NECs (10 months). Differences were noted in survival according to primary site with rectum and appendix (more than 30.0 years) having the longest median OS and NENs in the pancreas (7.0 years) and lung (4.5 years) with the shortest median OS (log-rank *P* < .001 for all).

**Figure 3.**
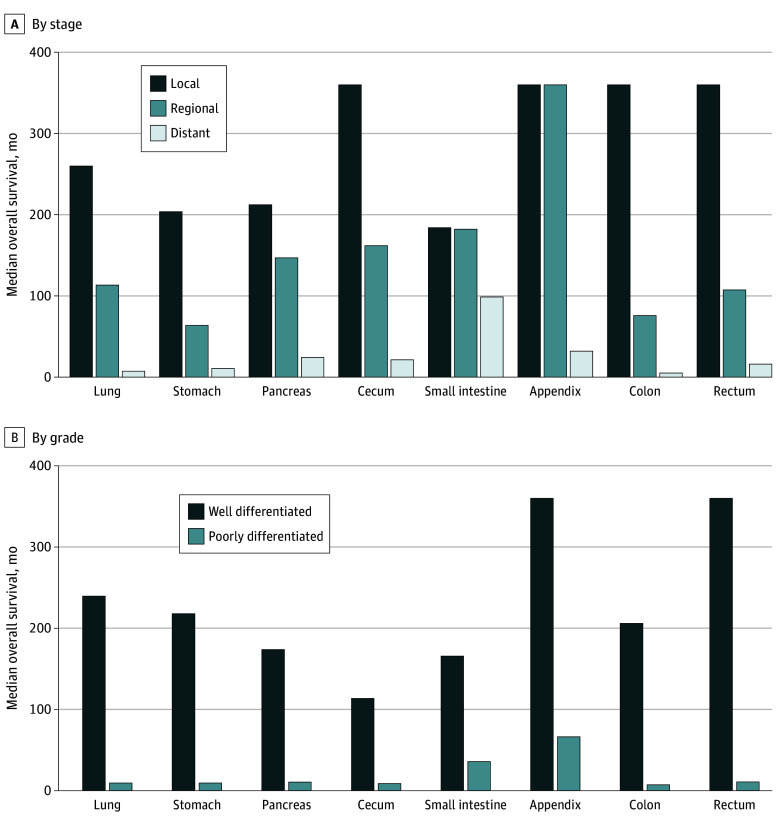
Median Overall Survival of Neuroendocrine Neoplasms

Because the proportions of advanced disease and higher grade NENs such as NECs at each primary site are variable and will have substantial impact on outcomes, we further analyzed these trends. We first evaluated survival patterns by primary site according to stage (Figure 3A). In localized NENs, median OS ranged from 15.3 years for small intestine to more than 30 years for the appendix, cecum, colon, and rectum. For regional NENs, median OS ranged from 6.3 years with a colon primary site to more than 30 years for the appendix. For distant NENs, those in the small intestine had the longest median OS (8.2 years) while those in the lung (7.4 months) and colon (5.1 months) had the shortest median OS, regardless of site. All these differences in OS were significant (log-rank *P* < .001).

We also examined the association of site and grade with OS (Figure 3B). Irrespective of site, patients with poorly differentiated NECs had poor OS, ranging from 35.7 to 66.2 months for the small intestine and appendix, respectively, down to 9.3 months 7.5 months for the colon (*P* < .001).

Finally, to provide useful point of care prognostic information, we estimated the median, 3-year, 5-year, and 10-year survival rates for well differentiated NETs in the SEER 18 cohort according to stage (2000-2021) (eTable 3 in Supplement 1). For localized and regional NETs, the median OS was not reached; 10-year survival rates ranged from 65.5% for the small bowel to 89.6% for the appendix, and 52.5% for colon and 90.3% for the appendix, respectively. For distant stage disease, the median OS for all sites was 6.7 years, ranging from 33.7 months at stomach to 102.9 months at small bowel.

### Multivariate Analysis of OS

Three multivariable models were constructed using SEER 17 data for 2000 to 2021: model 1 was the overall SEER 17 NEN cohort; models 2 and 3 were subsets that only included distant gastrointestinal and distant pancreatic NENs respectively. These models evaluated mortality during 2000 to 2006, 2007 to 2013, and 2014 to 2021 (the reference group).

#### Model 1

In the overall SEER 17 cohort (n = 131 100), patients diagnosed in the earlier time frames had an increased risk of mortality (2000-2006: HR, 1.42; 95% CI, 1.38-1.45; 2007-2013: HR, 1.10; 95% CI, 1.08-1.13) compared with those diagnosed between 2014 and 2021. Patients with poorly differentiated NECs (HR, 2.26; 95% CI, 2.21-2.32) had over twice the hazard rate compared with those diagnosed with well-differentiated NETs. Patients with distant metastases had nearly 3 times the hazard rate of death compared with patients with localized disease (HR, 2.96; 95% CI, 2.90-3.02). Patients in the oldest age group (older than 60 years) had over 6 times the hazard rate of mortality compared with the youngest age group (HR, 6.31; 95% CI, 5.67-7.03). Other covariates significantly inversely associated with survival included racial groups that were not Asian or Pacific Islander and primary sites that were not the lungs (Table 2).

**Table 2.** Multivariate Survival Analysis of Neuroendocrine Neoplasm Patients Diagnosed, 2000-2021

Covariate	HR (95% CI)		
	Model 1: Total SEER 17 NET cohort (n = 131 100)	Model 2: Distant gastrointestinal NET (n = 8386)	Model 3: Distant pancreatic NET (n = 5003)
Year			
2014-2020	1 [Reference]	1 [Reference]	1 [Reference]
2000-2006	1.42 (1.38-1.45)	1.08 (0.99-1.18)	1.24 (1.12-1.38)
2007-2013	1.10 (1.08-1.13)	1.02 (0.96-1.08)	1.09 (1.01-1.17)
Grade			
1 and 2: Well differentiated	1 [Reference]	1 [Reference]	1 [Reference]
3 and 4: Poorly differentiated and undifferentiated; anaplastic	2.26 (2.21-2.32)	3.97 (3.72-4.24)	1.91 (1.71-2.12)
Race			
White	1 [Reference]	1 [Reference]	1 [Reference]
American Indian and Alaska Native	1.26 (1.13-1.40)	1.25 (0.88-1.77)	1.24 (0.84-1.83)
Asian or Pacific Islander	0.97 (0.93-1.01)	1.24 (1.08-1.42)	1.04 (0.91-1.18)
Black	1.19 (1.16-1.23)	1.12 (1.03-1.21)	1.19 (1.08-1.33)
Age, y			
≤30	1 [Reference]	1 [Reference]	1 [Reference]
31-60	2.62 (2.35-2.92)	1.04 (0.74-1.47)	0.83 (0.64-1.10)
≥61	6.31 (5.66-7.03)	1.88 (1.33-2.65)	1.43 (1.10-1.86)
Stage			
Localized	1 [Reference]	NA	NA
Regional	1.06 (0.99-1.04)		
Distant	2.96 (2.90-3.02)		
Site			
Lung	1 [Reference]	NA	NA
Appendix	0.43 (0.41-0.45)		
Cecum	0.65 (0.62-0.69)		
Colon	0.70 (0.68-0.74)		
Pancreas	0.70 (0.68-0.72)		
Rectum	0.28 (0.27-0.29)		
Small intestine	0.50 (0.49-0.51)		
Stomach	0.60 (0.58-0.63)		

Abbreviations: HR, hazard ratio; NA, not applicable; NET, neuroendocrine tumor; SEER, Surveillance, Epidemiology, and End Results

#### Models 2 and 3

In models 2 (distant gastrointestinal NENs, 8386 cases) and 3 (distant pancreatic NENs, 5003 cases), patients diagnosed more recently had better outcomes compared with earlier time frames. In these 2 models, the HR for 2000 to 2006 was 1.08 (95% CI, 0.99-1.18) and 1.24 (95% CI, 1.12-1.38) respectively compared with 2014 to 2021. The other covariates found to be significantly inversely associated with survival were poorly differentiated NECs (model 2: HR, 3.97 [95% CI, 3.72-4.24]; model 3: 1.91 [95% CI, 1.71-2.12]), African American race (model 2: HR, 1.12 [95% CI, 1.03-1.21]; model 3: 1.19 [95% CI, 1.08-1.33]), and the group aged over 60 years (model 2, HR, 1.88 [95% CI, 1.33-2.65]; model 3: HR, 1.43 [95% CI, 1.10-1.86]). In the distant gastrointestinal NEN model, Asian race (HR, 1.24 [95% CI, 1.08-1.42]) also had significantly higher rates for survival (Table 2). The *P* values for all significant comparisons were <.001.

## Discussion

The age-adjusted annual incidence of NENs has continued to increase in contrast to the incidence of all cancers that has been slowly down-trending. A transient dip in incidence noted in 2020 was likely because of the COVID-19 pandemic hampering timely diagnosis and reporting. In continuation of our previously noted findings, we again show that survival of distant stage NENs has improved, likely reflecting continued improvements in therapeutic options.

This rise in rates of incidence continue to be most marked for localized stage and for well differentiated NETs likely due to increased incidental diagnosis of these neoplasms during evaluation for other, unrelated conditions. A 2019 study^16^ evaluating cancer epidemiological trends aimed to develop signatures based on relative rates of incidence of overall, metastatic, and mortality trends. A cancer where there has been an increase in overall incidence over time with relatively stable incidence of metastatic disease suggests overdiagnosis of early stage, indolent cases that may be otherwise clinically insignificant. Our study suggests that the epidemiology of NETs largely fits this pattern, highlighting the need for research into optimal management early stage disease whose incidence rates have risen the most concomitant with those in younger patients. For instance, in the case of appendix NETs, one of the most common NETs, the median age of diagnosis was 49 years as compared with the entire cohort at 62 years. Furthermore, most of these patients tend to have well differentiated locoregional tumors. Recently, in contrast to the traditional approach, right hemicolectomy has not been shown to be of benefit in patients with appendix NETs with 1 to 2 cm.^17,18^ It is also critical to identify biomarkers that may portend recurrent or metastatic spread for more aggressive management with resection and additional postsurgical adjuvant therapies as being explored by the ongoing SWOG S2104 study for high-risk pancreatic NETs (NCT05040360).

Starting in 2018, the SEER registries incorporated Ki-67 and mitotic index for classification of NENs. These early data suggest that G1, G2, and G3 NENs account for 75%, 16%, and 9%, respectively, with the most common GEP sites for G3 NENs being colorectal, pancreas, and appendix. In addition, 6% of all NENs are poorly differentiated. However, significant confusion persists. During our analysis, we found that lung NENs were being classified utilizing GEP NEN criteria. Furthermore, it is not currently possible to delineate G3 NENs into well- vs poorly differentiated NENs as outlined in the latest World Health Organization classification as the SEER program acquires these data only when grade information based on Ki-67 and/or mitotic index is unavailable. Nevertheless, as these deficiencies are addressed in the future, we anticipate that the SEER registries will provide even more valuable information.

The prevalence of NENs has continued to rise—the 20-year adjusted limited duration prevalence for the US population for January 1, 2021, was estimated to be 248 546 persons, significantly higher than what was reported for 2017 at 171 321 persons.^2^ However, this number includes all patients with a diagnosis of NEN in the past 20 years before the date of estimate irrespective of whether they have active disease or not. Given that the majority of NENs tend to be early stage disease, most likely had a diagnosis of localized or locoregional disease that was resected. Section 526(a)2(A) of the Food, Drug and Cosmetic Act defines a rare disease or a condition with prevalence less than 200 000 persons in the US and prevalence as the number of persons in the US who have been diagnosed as having the disease or condition at the time of the submission of the request for orphan-drug designation.^19^ A drug developed for such an indication is designated as an orphan drug that provides substantial assistance and incentives. NEN are heterogenous having different molecular signatures, prognosis, and patterns of response to therapy. Because most drug development occurs for NEN patients with advanced disease of subsets such as pancreatic NETs, GEP-NETs, or extra-pancreatic NETs who will form only small proportions of all NEN, most indications will continue to fulfill the criteria for orphan drug designation.

Our study also shows improvement in survival for patients with NENs over time, including those with advanced disease. Some of these improvements may be related to stage migration but also likely reflects the continual progress in the therapeutics for NETs that now include somatostatin receptor targeted imaging and therapies, novel targeted agents and in select cases cytotoxic therapies.^20,21^ Given that several of these are recent developments these improvements in survival in an indolent tumor type like NETs are likely are an underestimation of their long-term positive impact. Although race and gender have been shown to be prognostic, the underlying mechanisms are not fully understood and should be explored further. Also, every attempt should be made to ensure diversity and adequate representation of patients in clinical trials and when not possible to consider follow-up postapproval studies.

### Limitations

Our study had several limitations. The SEER database has only recently incorporated Ki-67 and mitotic index for NENs, and thus these data were too early to be fully incorporated. Second, other key determinants of outcomes such as prognostic factors, treatment histories, and functional status are not captured in the SEER database. Third, it is well known that NETs may be underreported thus leading to underestimations of their epidemiology.

## Conclusions

In this repeated measures cross-sectional analysis, we show that the incidence and prevalence of NENs continue to rise in association with increased diagnosis of early-stage disease. Survival for distant stage NENs has also improved over time with age, race, gender, histologic grade, primary site, and stage at diagnosis associated with survival.

## Notes

Supplement 1. **eTable 1.** Incidence Rates of Neuroendocrine Neoplasms over Time Including Age Specific Incidence Rates **eTable 2. **10-Year and 20-Year Prevalence Counts and Rates of Neuroendocrine Neoplasms **eTable 3.** Median Survival of Distant Stage Grade 1, 2 Neuroendocrine Tumors Diagnosed 2000-2021 **eFigure.** Annual Age-Adjusted Incidence of NEN by Stage and Differentiation

Supplement 2.
Data Sharing Statement
