# Errors in Abstract and Figure 2

**DOI:** 10.1001/jamanetworkopen.2025.47077

**Published:** 2025-11-05

In the Original Investigation titled “Epidemiology of Neuroendocrine Neoplasms in the US,”^1^ published June 24, 2025, the estimated 20-year limited duration prevalence of neuroendocrine neoplasms in the US on January 1, 2021, should have been reported as 248 546 cases instead of 243 896. In addition, the decimal placement in the y-axes of Figure 2 was incorrect. This article has been corrected.^1^
