# Supplementary material for: Epidemiology of Neuroendocrine Neoplasms in the US
Source: JAMA Netw Open. 2025 Jun 24;8(6):e2515798. doi: 10.1001/jamanetworkopen.2025.15798 (PMC12188367; doi:10.1001/jamanetworkopen.2025.15798)
Supplement: Supplement 1. — eTable 1. Incidence Rates of Neuroendocrine Neoplasms over Time Including Age Specific Incidence Rates eTable 2. 10-Year and 20-Year Prevalence Counts and Rates of Neuroendocrine Neoplasms eTable 3. Median Survival of Distant Stage Grade 1, 2 Neuroendocrine Tumors Diagnosed 2000-2021 eFigure. Annual Age-Adjusted Incidence of NEN by Stage and Differentiation [file jamanetwopen-e2515798-s001.pdf]

Supplemental Online Content

Dasari A, Wallace K, Halperin DM, et al. Epidemiology of neuroendocrine neoplasms in the United States. *JAMA Netw Open.* 2025;8(6):e2515798. doi:10.1001/jamanetworkopen.2025.15798

eTable 1. Incidence Rates of Neuroendocrine Neoplasms over Time Including Age Specific Incidence Rates

eTable 2. 10-Year and 20-Year Prevalence Counts and Rates of Neuroendocrine Neoplasms

eTable 3. Median Survival of Distant Stage Grade 1, 2 Neuroendocrine Tumors Diagnosed 2000-2021

eFigure. Annual Age-Adjusted Incidence of NEN by Stage and Differentiation

This supplemental material has been provided by the authors to give readers additional information about their work.

© 2025 Dasari A et al. *JAMA Network Open*.

eTable 1. Incidence of Neuroendocrine Tumors (NETs) Over Time

					Age specific incidence		
Registry	Year	Rate	Number of NET cases	Number at risk	less than 50	50-64 years	65 or older
SEER 8	1975	1.64	234	16,366,979	0.78	2.96	5.05
	1976	1.43	209	16,559,968	0.60	2.95	4.35
	1977	1.67	243	16,756,321	0.69	3.06	5.60
	1978	1.43	221	16,973,780	0.55	2.60	5.10
	1979	1.69	256	17,220,419	0.57	3.52	5.90
	1980	1.35	212	17,497,895	0.47	2.95	4.46
	1981	1.38	214	17,726,075	0.49	2.79	4.75
	1982	1.57	249	17,908,438	0.44	3.27	5.97
	1983	1.63	264	18,119,311	0.48	3.67	5.81
	1984	1.63	269	18,322,036	0.46	3.52	6.12
	1985	1.63	274	18,537,281	0.54	3.54	5.64
	1986	2.53	423	18,738,412	0.81	5.39	8.98
	1987	2.57	437	18,951,274	0.78	5.63	9.21
	1988	2.64	458	19,202,290	0.80	5.39	9.90
	1989	2.88	507	19,441,008	0.86	5.94	10.83
	1990	2.69	483	19,744,594	0.82	5.30	10.31
	1991	3.05	561	20,057,672	0.87	6.46	11.46
SEER 12	1992	3.15	879	31,827,056	0.91	6.71	11.77
	1993	3.20	909	32,221,600	0.90	6.91	11.94
	1994	3.31	951	32,512,862	0.94	6.82	12.73
	1995	3.93	1,136	32,825,366	0.85	8.82	15.74
	1996	4.01	1,177	33,194,014	1.00	9.07	15.19
	1997	4.17	1,251	33,644,241	1.13	8.38	16.58
	1998	4.61	1,405	34,097,710	1.07	9.02	19.62
	1999	4.64	1,447	34,510,826	0.98	10.70	18.42
SEER 17	2000	4.83	3,374	74,952,547	1.23	10.36	18.92
	2001	4.61	3,280	75,823,654	1.22	9.82	17.78
	2002	5.09	3,703	76,597,496	1.29	11.47	19.32
	2003	5.22	3,865	77,323,962	1.36	10.89	20.61
	2004	5.42	4,096	78,044,525	1.39	11.95	20.76
	2005	5.53	4,248	78,545,195	1.39	12.00	21.56
	2006	5.93	4,627	79,131,483	1.47	12.71	23.43
	2007	6.15	4,925	79,874,119	1.52	13.80	23.57
	2008	6.29	5,151	80,716,323	1.54	14.40	23.86
	2009	6.40	5,365	81,524,852	1.63	14.47	24.14
	2010	6.58	5,645	82,281,126	1.72	14.65	24.86
	2011	6.66	5,794	82,934,170	1.73	14.32	25.76
	2012	7.13	6,351	83,540,674	1.88	15.73	27.02

© 2025 Dasari A et al. *JAMA Network Open*.

2013	7.36	6,701	84,115,242	1.99	15.95	27.92
2014	7.67	7,121	84,716,416	2.14	17.00	28.30
2015	8.12	7,615	85,323,181	2.77	17.26	27.94
2016	8.51	8,115	85,893,135	2.82	17.85	29.97
2017	8.49	8,185	86,367,466	2.95	17.77	29.15
2018	8.34	8,164	86,696,849	2.89	17.39	28.81
2019	8.46	8,395	86,912,013	2.92	18.15	28.69
2020	7.41	7,445	87,000,867	2.68	15.37	25.06
2021	8.52	8,613	87,479,595	3.15	17.61	28.44

© 2025 Dasari A et al. *JAMA Network Open*.

eTable 2. 10-Year and 20-Year Prevalence of NET

Year	20-year duration Prevalence	20-year Count	10-year duration Prevalence	10-year Count
2001	0.0075%	7,483		
2002	0.0141%	14,007		
2003	0.0201%	20,025		
2004	0.0257%	25,634		
2005	0.0307%	30,694		
2006	0.0349%	35,054		
2007	0.0387%	38,929		
2008	0.0421%	42,432		
2009	0.0450%	45,463		
2010	0.0477%	48,344		
2011	0.0501%	50,886	0.0075%	7,486
2012	0.0523%	53,168	0.0141%	14,014
2013	0.0543%	55,260	0.0201%	20,038
2014	0.0561%	57,121	0.0257%	25,653
2015	0.0576%	58,767	0.0307%	30,717
2016	0.0590%	60,237	0.0350%	35,081
2017	0.0603%	61,513	0.0387%	38,958
2018	0.0614%	62,688	0.0421%	42,465
2019	0.0623%	63,634	0.0450%	45,498
2020	0.0631%	64,481	0.0478%	48,381
2021	0.0776%	67,999	0.0600%	52,579

© 2025 Dasari A et al. *JAMA Network Open*.

eTable 3. Median Survival and 3, 5, and 10-year Survival Percentages

	Localized				Regional				Distant			
	Median OS	3 YR%	5 YR%	10 YR%	Median OS	3 YR%	5 YR%	10 YR%	Median OS	3 YR%	5 YR%	10 YR%
**ALL PRIMARIES**	**NR**	**94.2**	**90.3**	**80.1**	**NR**	**90.7**	**84.9**	**70.4**	**80.28**	**68.9**	**57.0**	**38.4**
Lung	NR	94.7	90.6	79.5	NR	88.9	82.1	68.7	44.62	53.2	45.5	30.5
Pancreas	NR	93.5	90.0	78.3	NR	88.1	80.8	63.0	57.59	64.1	49.7	30.3
Stomach	NR	90.8	85.1	74.9	NR	84.8	73.6	56.4	33.73	49.6	37.6	28.2
Cecum	NR	93.2	87.0	77.7	NR	90.0	83.9	69.2	86.14	71.0	59.8	40.3
Rectum	NR	97.3	95.2	88.5	NR	90.7	84.6	62.7	39.71	53.2	34.7	15.4
Colon	NR	96.4	91.3	83.5	NR	79.5	68.5	52.5	26.00	42.9	31.4	20.3
Small Intestine	NR	87.4	80.1	65.5	NR	92.6	87.5	73.3	102.9	83.6	72.2	51.7
Appendix	NR	97.3	95.5	89.6	NR	96.8	95.2	90.3	71.20	65.2	54.4	32.3

NR = OS was greater than or equal to 360 months

© 2025 Dasari A et al. *JAMA Network Open*.

eFigure. Annual Age-Adjusted Incidence of NEN by Stage and Differentiation

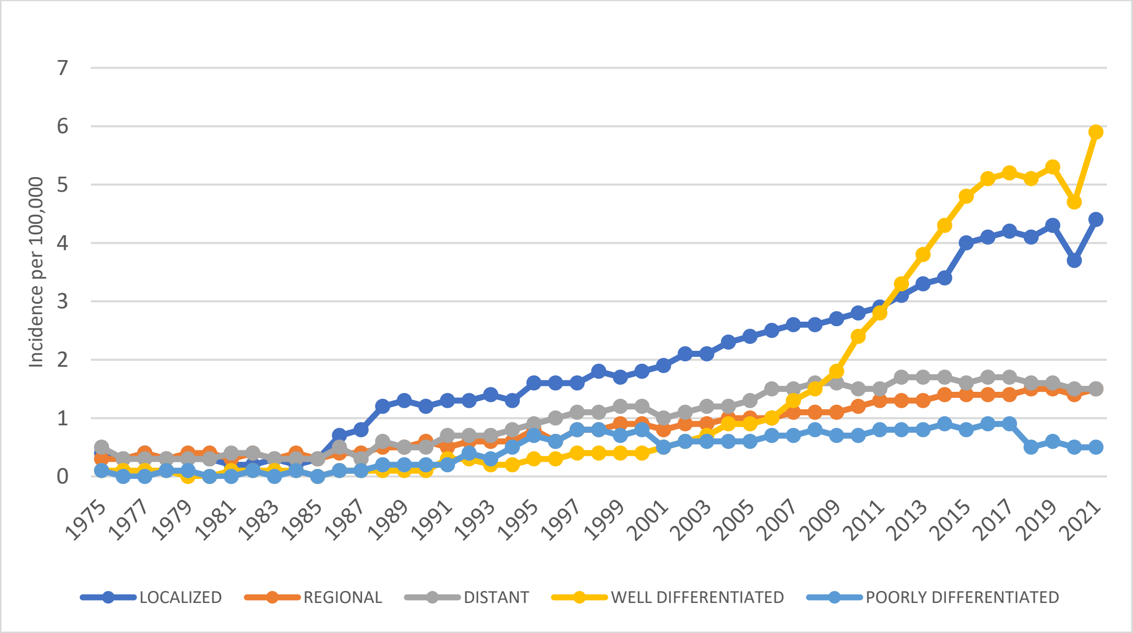

© 2025 Dasari A et al. *JAMA Network Open*.
