# Supplementary material for: Epidemiology of Neuroendocrine Neoplasms in the US
Source: JAMA Netw Open. 2025 Jun 24;8(6):e2515798. doi: 10.1001/jamanetworkopen.2025.15798 (PMC12188367; doi:10.1001/jamanetworkopen.2025.15798)
Supplement: Supplement 2. — Data Sharing Statement [file jamanetwopen-e2515798-s002.pdf]

Data Sharing Statement

Dasari. Epidemiology of Neuroendocrine Neoplasms in the US. *JAMA Netw Open*. Published June 13, 2025. doi:10.1001/jamanetworkopen.2025.15798

Data
Data available: Yes
Data types: Other (please specify), Data dictionary
Additional Information: SEER Data and data dictionary
How to access data: SEER Data and data dictionary
When available: With publication

Supporting Documents
Document types: Statistical/analytic code
How to access documents: please contact the corresponding author at adasari@mdanderson.org
When available: With publication

Additional Information
Who can access the data: researchers whose proposed use of the data has been approved
Types of analyses: any non-commercial purpose
Mechanisms of data availability: after approval of a proposal
